# Hyperpolarization of Molecular Deuterium

**DOI:** 10.1002/anie.202521985

**Published:** 2026-03-08

**Authors:** Theresa L. K. Hune, Anakin Aden, Julius F. Matz, Denis Moll, Ilya Kuprov, Stefan Glöggler

**Affiliations:** ^1^ NMR Signal Enhancement Group Max Planck Institute For Multidisciplinary Sciences Göttingen Germany; ^2^ Center For Biostructural Imaging of Neurodegeneration of the University Medical Center Göttingen Göttingen Germany; ^3^ Division of Medical Physics Department of Radiology University Medical Center Freiburg Faculty of Medicine University of Freiburg Freiburg Germany; ^4^ Department of Chemical and Biological Physics Weizmann Institute of Science Rehovot Israel; ^5^ School of Chemistry and Chemical Engineering University of Southampton Southampton UK; ^6^ Advanced Imaging Research Center University of Texas Southwestern Medical Center Dallas Texas USA; ^7^ Department of Biomedical Engineering University of Texas Southwestern Medical Center Dallas Texas USA

**Keywords:** Deuterium, hyperpolarization, NMR, orthodeuterium, parahydrogen

## Abstract

Hyperpolarized deuterium provides a promising alternative to hyperpolarized hydrogen for molecular sensing in NMR, particularly in hydrogen‐rich environments where proton detection is hindered by strong background resonances. Using a homogeneous iridium catalyst (IrIMes) and nicotinamide as substrate, we demonstrate the generation and detection of hyperpolarized molecular deuterium. The resulting resonance exhibits a pronounced partially negative line (PNL), strongly enhanced compared to thermal deuterium signal, and reproducible by simulation. A transient PNL is further observed during the initial phase of catalyst activation, highlighting sensitivity to transient intermediates. Notably, the enhanced PNL only arises in the presence of nicotinamide, confirming sensitivity to the presence of the substrate. These findings establish hyperpolarized orthodeuterium as a viable molecular sensor capable of providing valuable spectroscopic information of catalytic hydrogen‐bound complexes. Together with recent observations of PNLs in aqueous media, our results underscore the potential of hyperpolarized molecular deuterium to probe catalytic and enzymatic cycles under biologically relevant conditions.

## Graphical abstract

Hyperpolarized molecular deuterium is generated and detected using a nonhydrogenative SABRE catalyst with nicotinamide. The resonance shows a strongly enhanced partially negative line, substrate‐dependent and sensitive to transient intermediates. This establishes hyperpolarized deuterium as a promising probe for catalytic hydrogen complexes and enzymatic processes, particularly useful in proton‐rich environments.

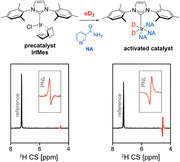

## 1 Introduction

Deuterium is a heavy isotope of hydrogen with a natural abundance of 0.015 %. In addition to the proton, its nucleus contains an unpaired neutron, resulting in the overall nuclear spin of *I*  =  1. Deuterated samples are useful in mass spectrometry [1, 2, 3], neutron scattering [4, 5], and magnetic resonance [6, 7, 8]. Swapping protons for deuterons improves resolution in macromolecular NMR spectroscopy and helps with the analysis of metabolic pathways. Deuterium has also recently been rediscovered as a tool in magnetic resonance imaging: deuterated metabolites and organ‐specific imaging agents are compatible with real‐time in vivo detection [9, 10, 11].

Hyperpolarization of nuclear spins offers additional advantages by boosting magnetic resonance signals by several orders of magnitude [12, 13]; it benefits neutron scattering too [14]. Here, we introduce the generation of hyperpolarized molecular deuterium using orthodeuterium as a polarization source and discuss its applications to identification of catalytic intermediates, biological NMR spectroscopy, and its potential further uses as a biological sensor.

Deuterium nuclei have three spin states: |1, ±1〉 and |1, 0〉. In the deuterium molecule D_2_, those states are coupled to give nine states: a singlet state |*S*〉, three triplet states |*T_k_* 〉 and five quintet states |*Q_k_* 〉. These states differ in the symmetry of their spin wavefunction: singlet and quintet have symmetric spin wavefunctions; triplet states are antisymmetric with respect to atom label permutations. Due to different rotation statistics, symmetric and antisymmetric states are individually stable at room temperature; singlet and quintet states are called orthodeuterium (oD_2_), triplet states are called paradeuterium (pD_2_). At room temperature, the thermal energy is well above the energy difference between these states, therefore they are almost equally populated. This results in a mixture with ortho:para ratio of 6:3 called “normal” deuterium (nD_2_). Upon cooling down, deuterium is enriched in the ortho state which has a lower energy. At 25 K, the fraction of oD_2_ is 95.7%.

Just like parahydrogen induced polarization (PHIP), this spin order can be used to enhance NMR signals of other molecules. This has been demonstrated by deuteration of 1,2‐dideuteropropionitrile with ortho‐enriched D_2_ using Wilkinson's catalyst [15], as well as several other unsaturated molecules using the standard PHIP catalyst 1,4‐Bis(diphenylphosphino)butane(1,5‐cyclooctadiene)Rh(I)tetrafluoroborate [16]. Hyperpolarized HD was observed in a reversible interaction of pH_2_ with Crabtree's catalyst in methanol‐d_4_ [17], and during the catalysis of hydrogen activating enzymes interacting with parahydrogen in water [18]. However, net Zeeman hyperpolarization in molecular deuterium has not so far been reported.

## 2 Results and Discussion

**FIGURE 1:**
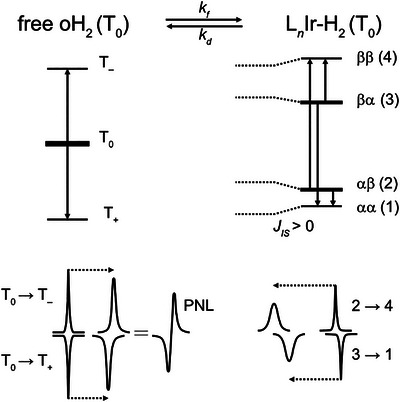
Energy level diagram explaining the emergence of PNL. Energy levels and transitions in free hyperpolarized oH_2_ and weakly‐coupled AX‐spin system are shown for the case of positive *J_IS_* in the complex Ir‐H_2_ (*I* and *S* denoting the two binding sites for hydrogen). The diagram describes the situation where singlet/triplet mixing at the catalyst has already taken place. Adapted from [19].

In the presence of certain catalysts, free parahydrogen can give rise to characteristic anti‐phase resonances called the partially negative line (PNL) [19, 20]. In recent years, this effect has been reported for several combinations of catalysts, substrates, and solvents [21, 22, 23, 24]. The specific pattern originates from the transition between para and ortho states at the catalyst and subsequent detection of hyperpolarized orthohydrogen exchanging between the free and the catalyst‐bound form. Free parahydrogen in solution is NMR silent due to its overall nuclear spin of 0. When parahydrogen binds to the catalyst and the two hydrogen nuclei exhibit different Larmor frequencies in the transient complex, the atom permutation symmetry is broken and the transition from |*S*〉 to |*T*_0_〉 becomes possible. After dissociation, this results in nonthermally populated orthohydrogen in the |*T*_0_〉 state. In absence of further perturbations, the resulting transitions |*T*_0_〉 to |*T*_−_〉 and |*T*_0_〉 to |*T*_+_〉 would cancel and the overpoplulation of |*T*_0_〉 would not lead to the detection of an enhanced hydrogen signal. However, the ongoing chemical exchange between free oH_2_ and catalyst‐bound H_2_ results in a small shift of resonance frequencies toward each other. Figure 1 depicts this in more detail, displaying possible transitions from the mainly populated states |*T*_0_〉 in oH_2_ as well as the Zeeman states |αβ〉 (2) and |βα〉 (3) in the hydrogen‐bound metal complex. The transition from |*T*_0_〉 to |*T*_−_〉 in the free oH_2_ exchanges with the transition from (2) to (4) in the bound form, while the transition from |*T*_0_〉 to |*T*_+_〉 exchanges with the transition from (3) to (1). The size of the resonance shift due to exchange depends on the chemical shift difference between the exchanging transitions. As these differences are unequal for the positive and negative transitions due to the coupling *J_IS_* between the two hydrogen atoms at the two binding sites *I* and *S* in the transient complex, the two transitions |*T*_0_〉 to |*T*_−_〉 and |*T*_0_〉 to |*T*_+_〉 are shifted by different amounts. This leads to an incomplete cancellation and the emergence of the PNL [19].

As this phenomenon has only been observed in hydrogen so far, we decided to investigate whether orthodeuterium could also be used to generate hyperpolarized molecular deuterium. We chose the [IrCl(COD)(IMes)] catalyst (IrIMes) dissolved in natural abundance benzene to follow reaction conditions in which the PNL had been observed for parahydrogen [20]. We used quadruple stoichiometric excess of nicotinamide and orthodeuterium gas pressure of 7 bar. Activation was performed by bubbling D_2_ through the sample for several minutes.

**FIGURE 2:**
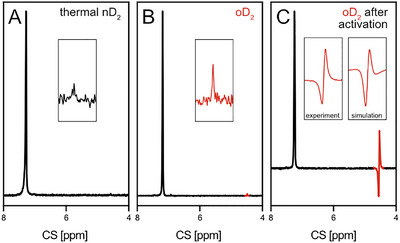
^2^H NMR spectra acquired (A) after passing thermal equilibrium nD_2_ through a solution of benzene‐h_6_ with 11% benzene‐d_6_; (B) after passing oD_2_ through a solution of benzene‐h_6_ with 11% benzene‐d_6_; (C) after passing oD_2_ through a solution of activated IrIMes (5 mM) and nicotinamide (NA, 20 mM) in benzene‐h_6_ with 11% benzene‐d_6_. Spectra shown in A and B were acquired following a 90° excitation pulse, whereas C was recorded using a 45° flip angle. The resonance at 7.3 ppm belongs to the reference benzene‐d_6_. The insets focus on the region of free D_2_ in the spectra. The inset in (C) also shows the simulated ^2^H resonance. Simulation details are given in the SI.

Figures 2A and B show ^2^H NMR spectra acquired following a 90° excitation pulse after passing nD_2_ and oD_2_ through a reference solution of benzene‐h_6_ with 11% benzene‐d_6_ without catalyst or nicotinamide. Only two resonances are visible, belonging to free deuterium at 4.5 ppm and benzene‐d_6_ at 7.3 ppm. The spectra show a small resonance from D_2_: unlike parahydrogen, oD_2_ has some quintet state which does have an NMR signal in the absence of a catalyst; this is visible in Figure 2B. Neither the signal of oD_2_, nor of nD_2_, shows a negative part.

**FIGURE 3:**
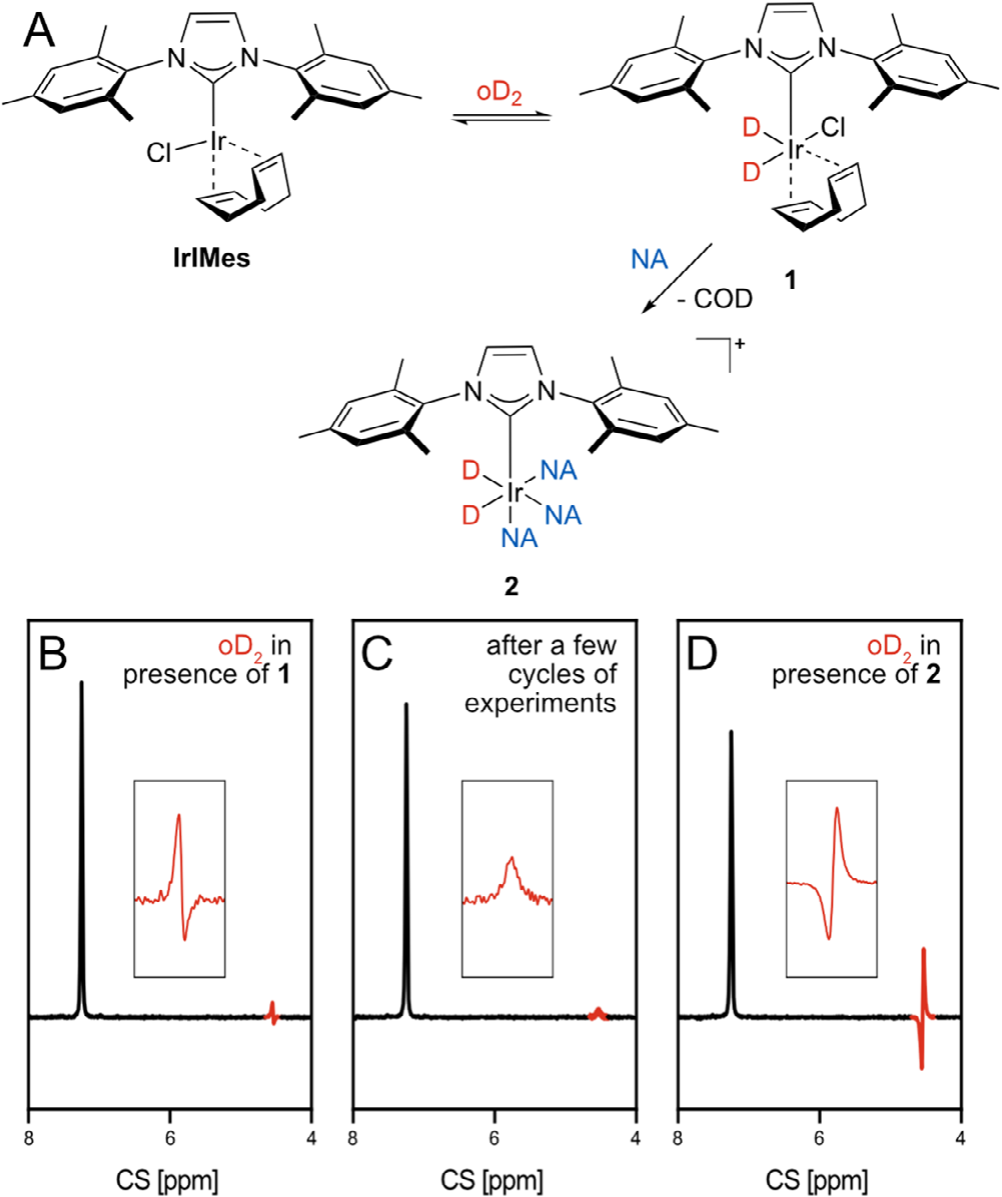
Scheme A shows the proposed reaction route during activation of the catalyst. Adapted from [20]. B‐D show recorded ^2^H NMR spectra of IrIMes (5 mM) and nicotinamide (20 mM) in benzene‐h_6_ with 11 % benzene‐d_6_ after repeated cycles of passing oD_2_ through the solution and acquisition of spectra. All spectra are recorded after application of an excitation pulse of 45°. B shows the spectrum after the first cycle, C shows a spectrum a few cycles later after disappearance of the PNL. The resonance of free D_2_ is colored in red. It changes to a small in‐phase singlet staying constant for several experiment cycles. Afterwards, the resonance abruptly increases and shows a PNL‐type signal again, shown in D. This shape stays for all consecutive cycles. The resonance at 7.3 ppm corresponds to the reference benzene‐d_6_. The insets focus on the region of free D_2_ in the spectra.

Figure 2C shows the ^2^H NMR spectrum after passing oD_2_ through the solution of activated IrIMes and nicotinamide in benzene. The resonance of free D_2_ is strongly enhanced when compared to the resonances of nD_2_ and oD_2_ and displays a PNL. The spectrum is recorded with a flip angle of 45°, as this maximizes the intensity of the PNL (for details on the flip angle dependence, please refer to the SI). The enhancement factor is around 86 compared to the thermal signal of nD_2_. Quantitative determination of the spin polarization for a PNL is not straightforward, as the observed resonance arises from the superposition of two signals of opposite phase that partially overlap and thus cancel each other. The signal enhancement only appears when both catalyst and the substrate are present in the solution, indicating that it is linked to the activated catalyst species **2** (Figure 3A), which forms after dissociation of the cyclooctadiene (COD) ligand. When using thermal equilibrium deuterium, the PNL signal does not appear. The second inset in Figure 2C shows the simulated ^2^H NMR spectrum in blue. It reproduces the shape of the experimentally observed resonance even with a minimal kinetics model in which the rate constants were chosen to match the experimental spectra. The signal inversion observed in the presence of nicotinamide is simulated by a reduced dissociation rate of deuterium at the catalyst, which is caused by the catalyst's activation. This results in stronger triplet transitions with the opposite phase, causing signal inversion. Simulation details are given in the SI.

In experiments with parahydrogen, a gradually decreasing PNL was observed during catalyst activation, while in‐phase hydrogen signals were observed for the activated complex. The PNL disappears quickly after hydrogenation of the sample due to the rapid para‐ortho conversion at the catalyst and return to equilibrium [20]. To investigate the evolution of the D_2_ signal during activation of the catalyst, a series of cycles of passing D_2_ through the sample and subsequent acquisition of spectra were performed. Scheme 3A shows the precatalyst IrIMes, as well as the proposed pathway for its reaction with oD_2_ and nicotinamide (NA) leading to the activated species with bound deuterium and nicotinamide **2**. Figure 3B–D show the recorded deuterium spectra after several cycles of the above‐described procedure. Figure 3B shows the spectrum from the first experiment cycle. It shows a small PNL at the chemical shift of free D_2_, the enhancement factor of this signal can be calculated to 13 compared to the thermal signal of nD_2_. In the second cycle, the PNL already starts to disappear, the spectrum showing a reduced negative part of the resonance. In the subsequent cycles, the resonance displays a small and broad singlet, which is displayed in Figure 3C. Suddenly, after several subsequent cycles, the intensity of the resonance increases again and changes to the PNL shape described above evoked by the activated complex, displayed in Figure 3D. This signal is much stronger than the initial PNL and is persistent for all consecutive cycles. It exhibits the opposite phase which we reproduced in simulations considering different exchange parameters (Figure S6 in SI). When conducting these experiments in the presence of the catalyst but in the absence of nicotinamide, the small initial PNL is still observable (Figure S2 in SI). This is similar to the results observed for parahydrogen, and links this effect to an early intermediate during catalyst activation **1**, in which the substrate is not yet bound [20]. The resonance of the reference benzene‐d_6_ appears to show a reduction in subsequent cycles. That is due to increasing B_0_‐inhomogeneity with each experiment cycle. The integral of the resonance remains constant.

In the case of parahydrogen, the activation of the catalyst can be followed by the appearance of hydride signals in the proton spectra [20]. In the experiments performed here, the only resonances observed belong to benzene‐d_6_ and free D_2_. No deuterides were observed because of their low signal‐to‐noise ratio.

The PNL has recently been observed in aqueous solution when parahydrogen was fed into the catalytic cycle of [Fe]‐hydrogenase [18]. This motivated our investigation into using ortho‐enriched deuterium as molecular sensor to report on catalytic activity. As it does not face signal obstruction by the dominant water peak, detection of weak resonances from transient species may be improved. The natural abundance of HDO in biological samples could act as an internal standard for signal quantification. Our experiments showed that, without catalytic assistance, oD_2_ exhibits no strong NMR signal. However, in the presence of the tested catalyst, a slight transient enhancement becomes detectable in the form of a PNL. A stronger resonance emerges only upon formation of the active catalyst species when a substrate, in this case nicotinamide, is introduced, highlighting the system's potential applicability as a selective biological sensor for different catalytic species. Nicotinamide's primary role is to stabilize the active complex and to alter the electronic structure of the iridium center. Consequently, the effect itself is not necessarily substrate‐specific; rather, the size and shape of the PNL are governed by ligand‐dependent binding and exchange kinetics. This is also the case for the PNL observed on hydrogen, as the work of Czarnota et al. showed: amplitude and phase of the PNL are influenced by the kinetics of the chemical exchange between the free and the bound form of D_2_ as well as the structure of the bound complex [20]. We, however, envision that substrate specific molecular probes can be designed that make use of the PNL effect. An indication how to approach this challenge is given by recent work of Tessari and coworkers [25]: The same metal complex as used in our studies shows different chemical shifts associated with hydrides depending on bound metabolites. We expect that this can serve as a method for obtaining substrate specific PNL patterns that may also be identifiable via hyperpolarized CEST experiments.

## 3 Conclusion

In conclusion, we have shown that hyperpolarized orthodeuterium can be generated and detected using a nonhydrogenative SABRE catalyst with nicotinamide. The resulting signal exhibits a pronounced partially negative line (PNL) and is significantly enhanced only in the presence of the substrate nicotinamide. In addition, a short‐lived PNL appears during the initial stage of catalyst activation, demonstrating that the approach is sensitive to transient intermediates. The sensitivity of the resonance toward substrate and exchange kinetics makes hyperpolarized deuterium a valuable tool for monitoring catalytic hydrogen‐bound complexes. Beyond the system presented here, the method offers a promising route for studying catalytic and enzymatic processes in aqueous environments, where traditional proton‐based detection is complicated by the strong water resonance.

## Conflicts of Interest

The authors declare no conflicts of interest.

## Supporting information




**Supporting File 1**: anie71730‐sup‐0001‐SuppMat.pdf.

## Data Availability

The data that support the findings of this study are available in the supplementary material of this article.
