# Supplementary material for: Hyperpolarization of Molecular Deuterium
Source: Angew Chem Int Ed Engl. 2026 Mar 8;65(16):e21985. doi: 10.1002/anie.202521985 (PMC13080423; doi:10.1002/anie.202521985)
Supplement: Supplementary file 1 — Supporting File 1: anie71730‐sup‐0001‐SuppMat.pdf. [file ANIE-65-e21985-s001.pdf]

SUPPLEMENTARY INFORMATION

Hyperpolarization of molecular deuterium

Theresa L.K. Hune [1,2,3], Anakin Aden [1,2], Julius F. Matz [1,2], Denis Moll[1,2], Ilya Kuprov [4,5], Stefan Glöggler [1,2,6,7]*

[a]NMR Signal Enhancement Group, Max Planck Institute for Multidisciplinary Sciences, Am Fassberg 11, 37077 Göttingen, Germany.

[b]Center for Biostructural Imaging of Neurodegeneration, University Medical Center, Von-Siebold-Str. 3 A, 37075 Göttingen, Germany.

[c]Division of Medical Physics, Department of Radiology, University Medical Center Freiburg, Faculty of Medicine, University of Freiburg, Kilianstr.5a, 79106 Freiburg

[d]Department of Chemical and Biological Physics, Weizmann Institute of Science, 234 Herzl Street, Rehovot 7610001, Israel

[e]School of Chemistry and Chemical Engineering, University of Southampton, Southampton SO171BJ, United Kingdom

[f]Advanced Imaging Research Center, University of Texas Southwestern Medical Center, Dallas, Texas 75390, USA.

[g]Department of Biomedical Engineering, University of Texas Southwestern Medical Center, Dallas, Texas 75390, USA.

**E-mail: stefan.gloeggler@UTSouthwestern.edu*

Contents

S1. Experimental procedures 2

S2. Pulse flip angle dependence 2

S3. Control experiments without nicotinamide 4

S4. Kinetics of the PNL Signal 4

S5. Inversion Recovery Experiments with oD_2 6

S6. ALTADENA type experiments 6
S7. Numerical simulations.. 7
References...11

S1. Experimental procedures

Ortho-enrichment of D_2 gas (99.9% purity, 99.8% isotope fraction) was performed using the same helium-cooled generator as the one used to make parahydrogen: Sumimoto HC-4A helium compressor, Sumimoto Cold Head CH-204 with a reaction chamber by ColdEdge Technologies with a Lake Shore Cryotronics temperature controller and other components previously described[1]. The enrichment was performed at 25 K, resulting in a theoretical ortho-fraction of 0.957. The generated oD_2 was used immediately by transferring it to the sample tube via a home-built valve and tubing system.

The precatalyst [IrCl(COD)(IMes)] (IrIMes, M = 640 g/mol) was synthesized according to the procedure reported by Blanchard *et al.*[2] For sample preparation, 5 mmol of IrIMes were dissolved in degassed benzene-h_6. This combination of catalyst and solvent was used because it is known to yield observable partially negative line (PNL) in the case of parahydrogen.[3] Nicotinamide (M = 122 g/mol) was added to the solution to a concentration of 20 mM. For each sample, 400 µL of the resulting solution was placed into a 5 mm NMR tube along with 50 µL of benzene-d_6 which was used for the magnetic field lock and as a chemical shift reference: its signal at 7.0 ppm does not overlap with the free deuterium signal at 4.5 ppm.

All NMR experiments were performed at room temperature using a Bruker Ultrashield Avance III HD spectrometer operating at a proton Larmor frequency of 300 MHz (B_0 = 7.05 Tesla) equipped with a 5 mm room temperature broadband observe probe (PA BBO 300S1 BB-H-D-5 Z). The sample was degassed and connected to the home-built tubing system.[1] At the start of each experiment, oD_2 was bubbled through the sample for 7 seconds at a pressure of 7 bar; after a settling delay of 1 second, a 45°-degree pulse was applied, and free induction decay acquisition was started.

S2. Pulse flip angle dependence

Signals generated by single-spin orders show a pulse flip angle dependence of $\sin\theta$, resulting in a maximum intensity at 90°, zero intensity at 180°, and a maximum with inverted phase at 270°. Double-quantum coherences in weakly coupled spin pairs exhibit $\sin 2\theta$ flip angle dependence, therefore showing maximum signal at 45°, no signal at 90°, and a signal with an inverted phase at 135°. Recording spectra with flip angles of 45°, 90° and 135° after passing D_2 through a solution of catalyst IrIMes (5 mM) and nicotinamide (20

mM) in benzene-(h_6,d_6) yields the spectra shown in Figure 1(A,B,C) for the initial catalyst state. Figure 1(D,E,F) show the same for the activated catalyst. The inversion of the signal from 45° to 135° confirms that the resonance is caused by a double-quantum coherence. The small signal excited by the 90° pulse results from a combination of probe B_1 field inhomogeneity (the flip angle is slightly different across the sample) and the unavoidable small amount of thermal equilibrium state deuterium in the sample.

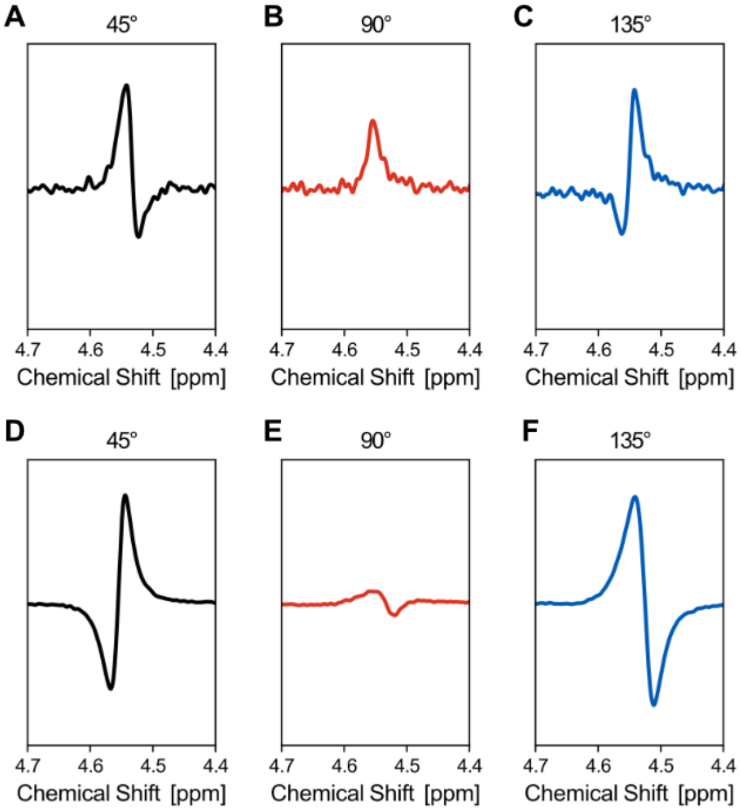

Figure S1. *Free deuterium NMR signals acquired after passing oD_2 through a solution of catalyst IrIMes (5 mM) and nicotinamide (20 mM) in benzene-(h_6,d_6) before activation (A-C) and after activation (D-F) of the catalyst. Pulse flip angle used for the excitation is indicated at the top of each panel. All spectra along one row are plotted in the same scale.*

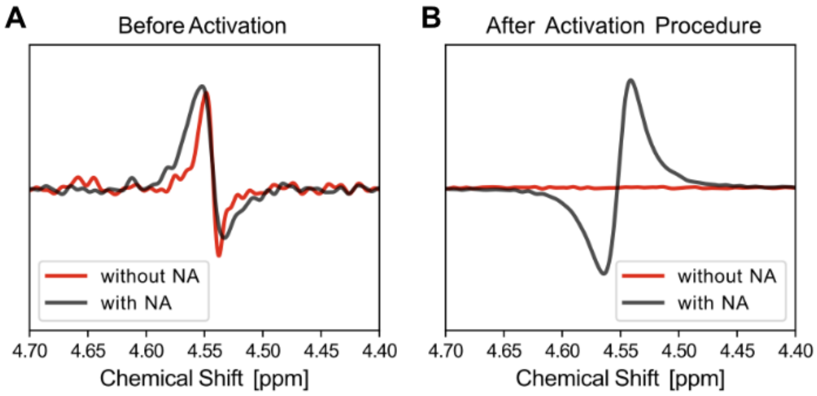

Figure S2. *Free deuterium NMR signals acquired after passing oD_2 through a solution of catalyst IrIMes (5 mM) with and without nicotinamide (NA) in benzene-(h_6,d_6) before (A) and after (B) activation of the catalyst. A 45° excitation pulse was used in both cases.*

S3. Control experiments without nicotinamide

When the experiments shown in Figure 3 of the main text are performed without nicotinamide, the initial partially negative line is still observable. However, the activation of the catalyst cannot occur due to the lack of the substrate. In this case, no significant free D_2 signal is visible: Figure S2 shows the excerpts from Figure 3 in black compared to the respective experiments conducted without nicotinamide in red.

S4. Kinetics of the PNL Signal

As mentioned previously, the PNL signal of the active complex is only obtained in the presence of nicotinamide and after bubbling with oD_2. To obtain information about the kinetic behavior of the PNL signal, a solution of IrIMes (5 mM) and nicotinamide (20 mM) was prepared in degassed in benzene-(h_6,d_6). Three aliquots of 450 µL of this solution were transferred to NMR tubes and used for experiments. After activation of the catalyst, a series of $\pi/4$-pulse detected single scan spectra were acquired with a variable delay after oD_2 bubbling for 7 s. Bubbling was conducted for each scan at a pressure of 7 bar. The experiment was repeated in triplicate for aliquots A and B while the experiment with aliquot C could only repeated in duplicate due to a decrease in sample activity. For each aliquot different variable delay lists were defined that were varied in an increasing, decreasing or alternating fashion for each repetition of the experiment.

The data was baseline corrected and phase corrected using Topspin 4.5.0. Further analysis of the acquired data was conducted using Python 3.0 within Jupyter notebook. The integral of the PNL was obtained by integrating the positive and negative part of the signal and adding the absolutes. For each aliquot the mean of the absolute PNL integral was calculated. The error was obtained from standard deviation using student factors for 90% prediction intervals. By plotting the PNL integral against the variable delay and fitting a monoexponential decay to the data the lifetime of the signal can be extracted (see Figure S3). A build-up phase for the PNL signal is visible for each sample. The maximum PNL

signal is obtained after 10.0 to 20.0 s depending on the sample. This value was chosen as the starting value for the exponential decay. Accordingly, the decay time τ of the PNL varied between 30 ± 3 s to 38 ± 3 s.

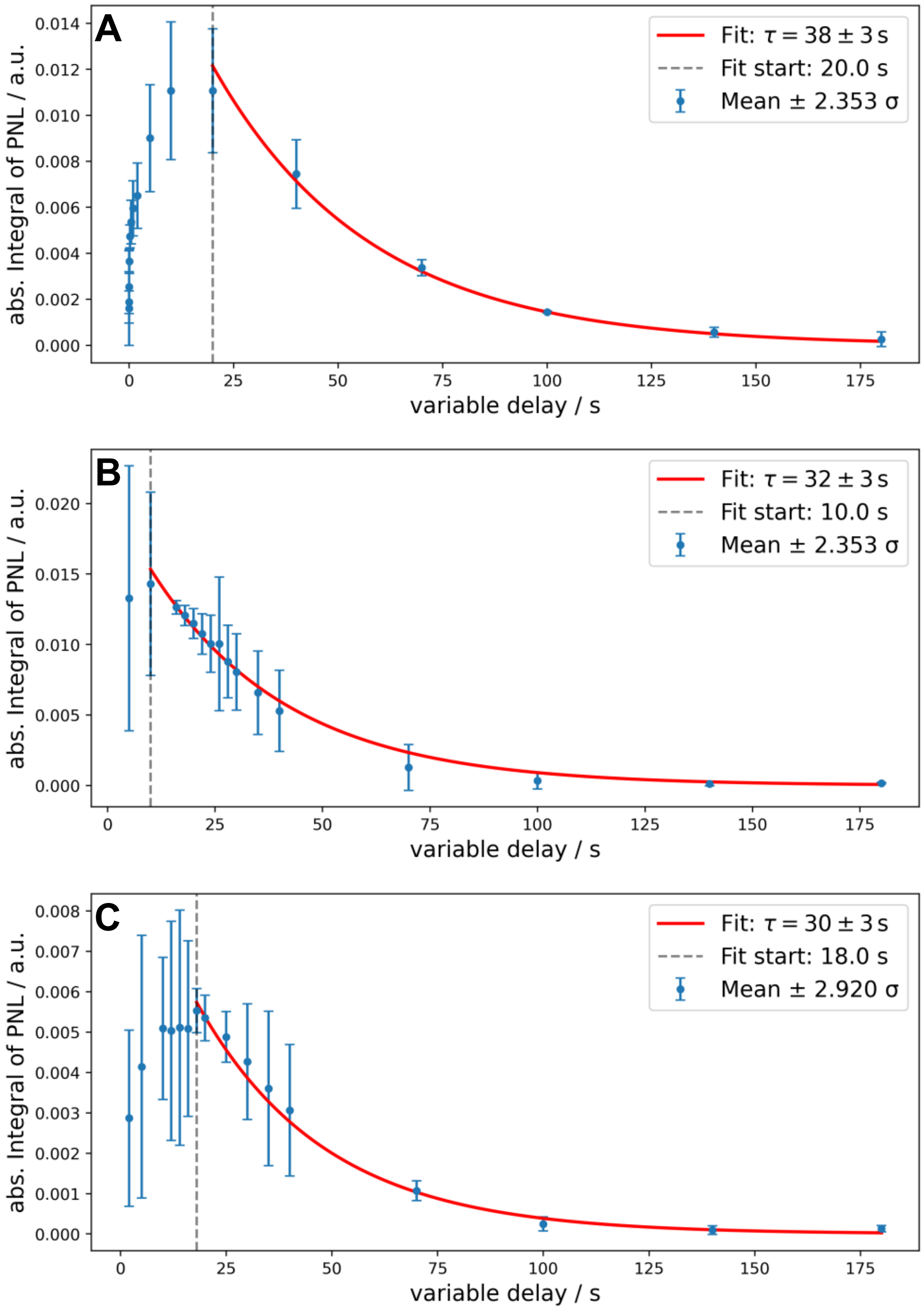

Figure S3. *Kinetics of D_2PNL signal. Experimental data points were obtained from the absolute integral of the PNL signal and fitted with a monoexponential decay function (* $I = I_0 * exp(-t/\tau)$*). ^2H NMR spectra were acquired at 7 T after passing oD_2 through a solution of catalyst IrIMes (5 mM) with nicotinamide (NA) in benzene-(h_6,d_6) for 7 s and* $\pi / 4$ *excitation pulse after a variable delay. Samples A & B were measured in triplicate while sample C was measured in duplicate due to decreasing activity.*

S5. Inversion Recovery Experiments with oD_2

To obtain information about the T_1 relaxation time of orthodeuterium in solution, inversion-recovery-type experiments were conducted. For this, 450 µL of degassed benzene-(h_6,d_6) were prepared for oD_2 bubbling experiments at 7 bar. For each acquisition, bubbling was conducted for 7 seconds before inversion recovery pulses were applied. The signals were averaged over eight scans. The data was baseline corrected and phase corrected using Topspin 4.5.0. For evaluation of the data, Python 3.0 within Jupyter notebook was used. The signal for oD_2 was integrated and plotted against the variable delay. Fitting of an exponential saturation curve to the measured data allowed the extraction of longitudinal relaxation times. The errors were obtained as standard deviation from the optimal fit parameters. The result is shown in Figure S4, analysis of these experiments yielded a T_1 time of oD_2 of 24 $\pm$ 1 s.

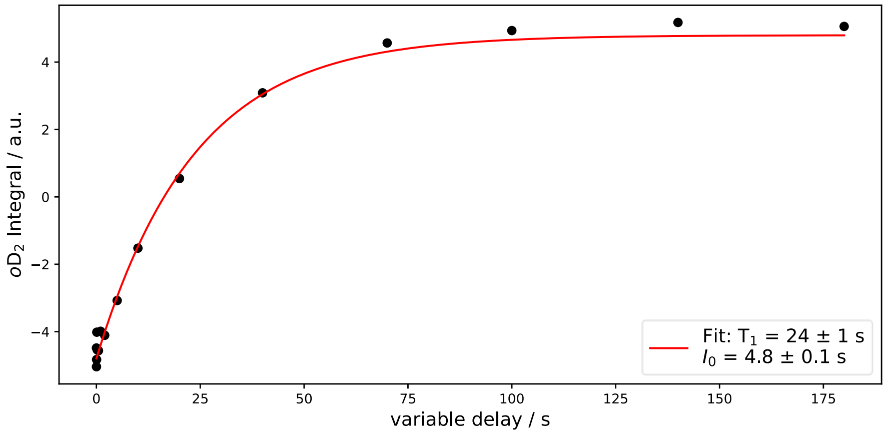

Figure S4. *Kinetics of oD_2 signal. Experimental data points were obtained from the absolute integral of the oD_2 signal and fitted with a exponential saturation function ($I = I_0 * \left(1-2*exp\left(-t/\tau\right)\right)$). NMR spectra were acquired at 7 T after passing oD_2 through benzene-(h_6,d_6) for 7 s.*

S6. ALTADENA type experiments

The behaviour of the PNL was tested when deuteration is conducted outside the magnet bore at earth magnetic field. For this experiment oD_2 was bubbled through an activated sample of IrIMes (5 mM) and nicotinamide (20 mM) in degassed benzene-(h_6,d_6). The bubbling was conducted for 7 s at a pressure of 7 bar using the same home-built bubbling setup as for all other experiments and the sample was inserted manually into the spectrometer after a delay time of 5 s, 10 s or 20 s. This configuration allowed a distance of around 2 m to the spectrometer, which was well outside out its 5G-line. The resulting spectra shown in Figure S5 were acquired with $\pi/4$ pulses.

All experiments independent of the shuttling delay exhibit the same behavior as experiments conducted at high-field with PNL signals for D_2 of the same phase as for the

activated Ir complex. The PNL intensity is the highest after a delay of 5 s and decreased with increasing delay.

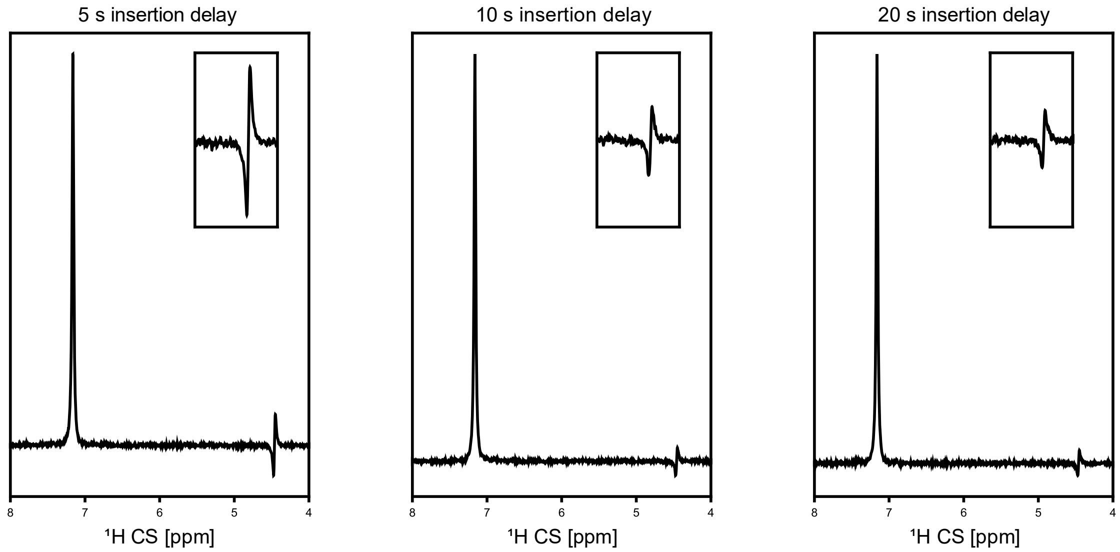

Figure S5. *^{2}H NMR signals acquired after passing oD_2 through a solution of catalyst IrIMes (5 mM) with nicotinamide (NA) in benzene-(h_6,d_6) under ALTADENA conditions and varying insertion delays. The spectra were acquired with 45° at 7 T.*

S7. Numerical simulations

All simulations were performed in *Matlab R2025b* using *Spinach*[4]. The minimal spin system that reproduces the observed behaviour contains four deuterons: a pair at 4.55 ppm corresponding to the dissolved gas and another pair at –13.5, –16.5 ppm corresponding to the bound deuterium molecule. In the former case, J_{DD} = 12 Hz, in the latter case J_{DD} = 0.24 Hz[5]. Quadrupolar interaction tensors for the bound deuterium molecule were estimated from a DFT calculation (M06/cc-pVDZ in SMD benzene in Gaussian16[6]) and used by the relaxation theory module of *Spinach*[7] to estimate the quadrupolar coupling contribution to the relaxation rates (Bloch-Redfield-Wangsness theory[8] with τ_C = 400 ps and all applicable cross-correlations[7,9]). Electronic structure theory calculation logs are included into the example set of Spinach (https://github.com/IlyaKuprov/Spinach) that also contains all Matlab code associated with this paper. For the activated catalyst bound deuterium molecules, the following quadrupolar coupling tensors were used:

$$\mathbf{Q}_{\mathrm{D1}} = 2\pi \begin{pmatrix} 108.2 & 0.1 & 28.1 \\ 0.1 & -55.6 & 4.5 \\ 28.1 & 4.5 & -52.6 \end{pmatrix} \cdot 10^3, \quad \mathbf{Q}_{\mathrm{D2}} = 2\pi \begin{pmatrix} -55.2 & 8.1 & -14.5 \\ 8.1 & -6.3 & -73.9 \\ -14.5 & -73.9 & 61.5 \end{pmatrix} \cdot 10^3 \qquad (1)$$

where the matrices enter the spin Hamiltonian as $\mathbf{S} \cdot \mathbf{Q} \cdot \mathbf{S}$ in which $\mathbf{S}$ is a vector of Cartesian spin projection operators. Dipole-dipole interaction tensors in the same reference frame were estimated from Cartesian coordinates (Angstroms) extracted from the optimised molecular geometry:

$$\mathbf{r}_{\mathrm{D1}}=\begin{pmatrix}-1.96 & 0.57 & -0.58\end{pmatrix},\quad \mathbf{r}_{\mathrm{D2}}=\begin{pmatrix}-0.18 & 1.40 & -1.63\end{pmatrix} \tag{2}$$

For the deuterium molecule bound to the pre-catalyst, the corresponding numbers are:

$$\begin{gathered}\mathbf{Q}_{\mathrm{D1}}=2\pi\begin{pmatrix}107.5 & 17.9 & -21.3\\ 17.9 & -49.7 & 2.0\\ -21.3 & 2.0 & -57.8\end{pmatrix}\cdot 10^3,\quad \mathbf{Q}_{\mathrm{D2}}=2\pi\begin{pmatrix}-52.7 & 9.1 & 43.8\\ 9.1 & -51.8 & 26.4\\ 43.8 & 26.4 & 104.5\end{pmatrix}\cdot 10^3\\ \mathbf{r}_{\mathrm{D1}}=\begin{pmatrix}-1.79 & 0.83 & 0.21\end{pmatrix},\quad \mathbf{r}_{\mathrm{D2}}=\begin{pmatrix}-0.61 & 0.78 & -1.51\end{pmatrix}\end{gathered} \tag{3}$$

For the dissolved deuterium gas in a specific rovibrational state, there is currently no reliable way to predict nuclear spin relaxation times *ab initio*, they were therefore set to empirically reasonable values of T_1 = 25 s, T_2 = 0.5 s within the extended T_1/T_2 approximation[9] used by *Spinach*.

The ortho-deuterium spin order for time-domain simulations was specified as described by the Bargon group[10] – the following elementary states:

$$\begin{gathered}\left|Q_{-2}\right\rangle=\left|\alpha\alpha\right\rangle,\quad \left|Q_{+1}\right\rangle=\frac{1}{\sqrt{2}}\left(\left|\alpha\beta\right\rangle+\left|\beta\alpha\right\rangle\right)\\ \left|Q_{0}\right\rangle=\frac{1}{\sqrt{6}}\left(\left|\alpha\gamma\right\rangle+2\left|\beta\beta\right\rangle+\left|\gamma\alpha\right\rangle\right)\\ \left|Q_{-1}\right\rangle=\frac{1}{\sqrt{2}}\left(\left|\beta\gamma\right\rangle+\left|\gamma\beta\right\rangle\right),\quad \left|Q_{+2}\right\rangle=\left|\gamma\gamma\right\rangle\\ \left|T_{+1}\right\rangle=\frac{1}{\sqrt{2}}\left(\left|\alpha\beta\right\rangle-\left|\beta\alpha\right\rangle\right),\quad \left|T_{-1}\right\rangle=\frac{1}{\sqrt{2}}\left(\left|\beta\gamma\right\rangle-\left|\gamma\beta\right\rangle\right)\\ \left|T_{0}\right\rangle=\frac{1}{\sqrt{2}}\left(\left|\alpha\gamma\right\rangle-\left|\gamma\alpha\right\rangle\right),\quad \left|S\right\rangle=\frac{1}{\sqrt{3}}\left(\left|\alpha\gamma\right\rangle-\left|\beta\beta\right\rangle+\left|\gamma\alpha\right\rangle\right)\end{gathered} \tag{4}$$

were projected into the irreducible spherical tensor basis set used by *Spinach*[11] and then assembled into the following density matrix

$$\boldsymbol{\rho}_{\mathrm{pump}}=\left|S\right\rangle\left\langle S\right|+\sum_{k=-2}^{2}\left|Q_k\right\rangle\left\langle Q_k\right| \tag{5}$$

Spinach was instructed to modify the kinetics superoperator as described by Kuprov and Hore[12] to pump this state continuously into the dissolved deuterium gas spin subsystem at a rate that was chosen to match experimental observations. The rest of the kinetics contained a pseudo-first order approximation of the association reaction:

$$\mathrm{D}_2+\mathrm{Cat}\underset{k_-}{\overset{k_+}{\rightleftarrows}}\mathrm{D-Cat-D} \tag{6}$$

in which the initial concentration of the catalyst is large enough and k_+/k_- ratio is small enough that the free catalyst concentration does not depend on time:

$$\begin{cases} \dfrac{d[\mathrm{D}_2]}{dt} = -k_+[\mathrm{D}_2][\mathrm{Cat}] + k_-[\mathrm{CatD}_2] \\ \dfrac{d[\mathrm{CatD}_2]}{dt} = k_+[\mathrm{D}_2][\mathrm{Cat}] - k_-[\mathrm{CatD}_2] \end{cases} \Rightarrow \begin{cases} \dfrac{d\left[\mathrm{D}_2^{\mathrm{free}}\right]}{dt} = -k_+^{\mathrm{eff}}\left[\mathrm{D}_2^{\mathrm{free}}\right] + k_-\left[\mathrm{D}_2^{\mathrm{bound}}\right] \\ \dfrac{d\left[\mathrm{D}_2^{\mathrm{bound}}\right]}{dt} = k_+^{\mathrm{eff}}\left[\mathrm{D}_2^{\mathrm{free}}\right] - k_-\left[\mathrm{D}_2^{\mathrm{bound}}\right] \end{cases} \quad (7)$$

in which $k_+^{\mathrm{eff}} = k_+[\mathrm{Cat}]$ and the right hand side is suitable for spin dynamics simulations because it is a first-order exchange reaction between different Hamiltonian and relaxation superoperator states of the same spin system. Values of $k_+^{\mathrm{eff}} = 1\ \mathrm{Hz}$ and $k_- = 5\ \mathrm{kHz}$ were empirically found to match experimental observations for the activated catalyst (Figure S6, right panel). A faster off rate of $k_- = 50\ \mathrm{kHz}$ is required to reproduce the experimental spectrum in the absence of nicotinamide (Figure S6, left panel).

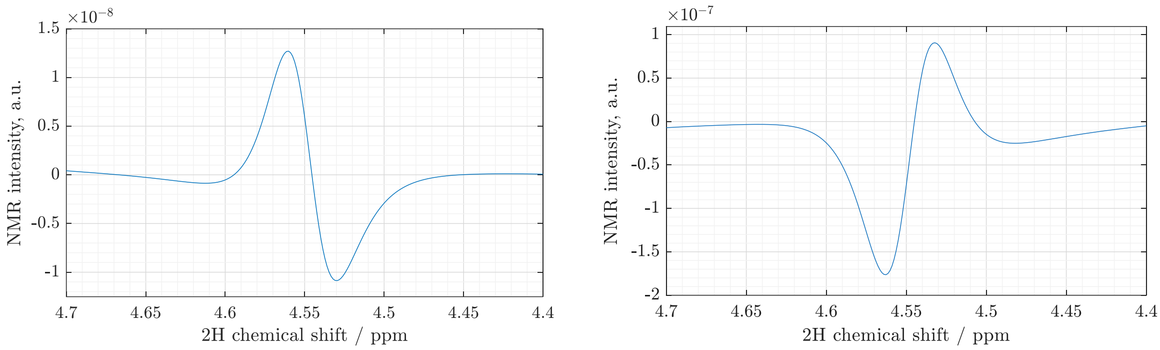

Figure S6. *Simulated ^{2}H spectra, showing the partially negative line after 7 seconds of oD_2 bubbling followed by a $(\pi/4)_Y$ pulse and acquisition under the continued action of the kinetics superoperator.* ***Left panel:*** *in the absence of nicotinamide (higher deuterium off rate).* ***Right panel:*** *in the presence of nicotinamide (lower deuterium off rate).*

Following successful simulations that demonstrated the expected dependence on all spin system and chemical kinetics parameters, we explore the spin system trajectory by projecting out pertinent states (Figure S7) and their coherences (Figure S8).

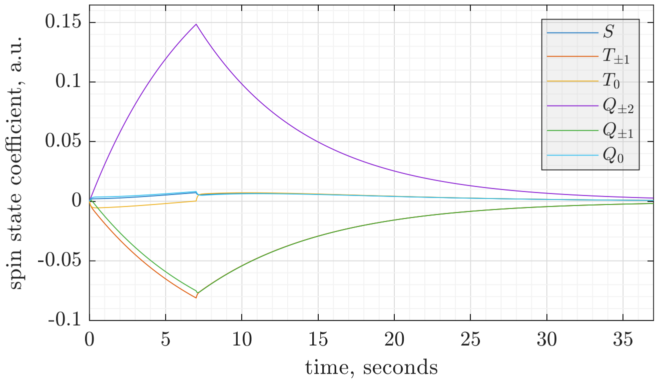

Figure S7. *Longitudinal basis state populations in a simulated experiment where 7 seconds of oD_2 bubbling are followed by 30 seconds of free evolution. The principal difference between the behaviour of symmetric (S, T_0, Q_0) and non-symmetric states is likely to be a consequence of their different relaxation and cross-relaxation behaviour under the NQI-driven relaxation superoperator in the presence of J-coupling and chemical shift difference during their residence time on the complex.*

During bubbling and shortly thereafter, the populations of the triplet states increase due to slow ortho-para conversion.

It is interesting to see which single-quantum transitions dominate the 45°-acquire spectrum at different values of the forward reaction rate – a significant difference could exist between protium and deuterium due to the kinetic isotope effect. Figure S8 shows the effect k_{+}^{eff} has on the transitions that make up the spectrum.

Signal phase is opposite for triplet and quintet transitions. As a result, the shape of the deuterium line is influenced significantly by the kinetic rate constants. The rates used to obtain Figure S6 were chosen to reproduce the experimental shape in Figure S2.

DFT calculation logs and *Matlab* code for all of the above are available in the example set of *Spinach* (https://github.com/IlyaKuprov/Spinach), including `deut_pair.m` function that returns the states of a spin-1 pair, classified by into singlet, triplet, and quintet.

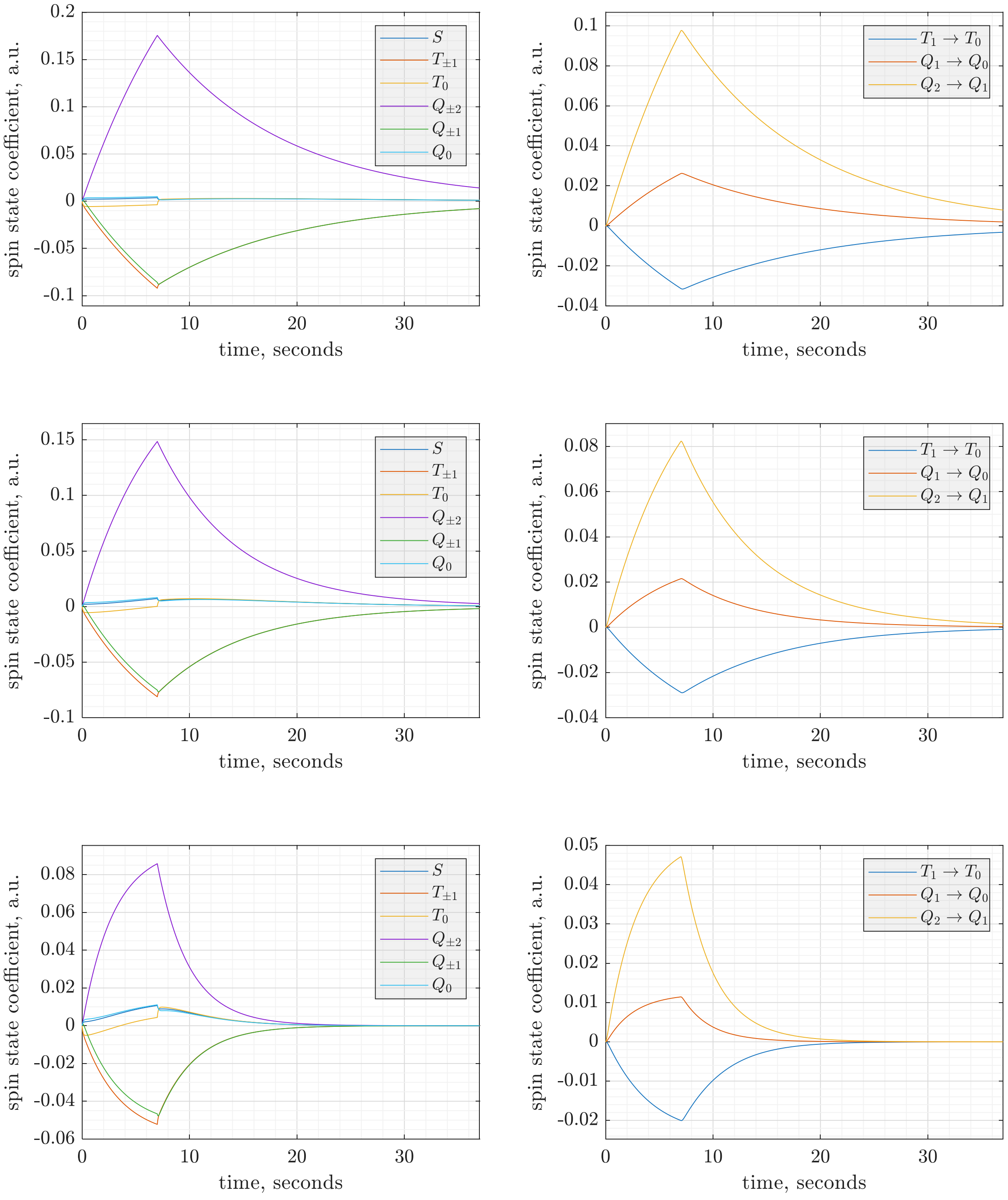

Figure S8. *Longitudinal basis state (left column, before the 45-degree pulse) and transverse coherence (right column, after the 45-degree pulse) population dynamics in a simulated experiment where 7 seconds of oD_2 bubbling are followed by 30 seconds of free evolution. Top row has k_+^{eff} = 0.25 Hz, middle row has k_+^{eff} = 1.00 Hz, and bottom row has k_+^{eff} = 4.00 Hz. Rapid spin relaxation in the bound state results in faster magnetisation decay as the residence time in the bound state is increased.*

References

(1) Ding, Y.; Korchak, S.; Mamone, S.; Jagtap, A. P.; Stevanato, G.; Sternkopf, S.; Moll, D.; Schroeder, H.; Becker, S.; Fischer, A.; et al. Rapidly Signal-enhanced Metabolites for Atomic Scale Monitoring of Living Cells with Magnetic Resonance. *Chemistry–Methods* **2022**, *2* (7), e202200023. DOI: https://doi.org/10.1002/cmtd.202200023.

(2) Blanchard, J. W.; Ripka, B.; Suslick, B. A.; Gelevski, D.; Wu, T.; Münnemann, K.; Barskiy, D. A.; Budker, D. Towards large-scale steady-state enhanced nuclear magnetization with in situ detection. *Magnetic Resonance in Chemistry* **2021**, *59* (12), 1208–1215. DOI: https://doi.org/10.1002/mrc.5161.

(3) Czarnota, M.; Mames, A.; Pietrzak, M.; Jopa, S.; Theiß, F.; Buntkowsky, G.; Ratajczyk, T. A Straightforward Method for the Generation of Hyperpolarized Orthohydrogen with a Partially Negative Line. *Angewandte Chemie* **2024**, *136* (12), e202309188.

(4) Hogben, H. J.; Krzystyniak, M.; Charnock, G. T.; Hore, P. J.; Kuprov, I. Spinach–a software library for simulation of spin dynamics in large spin systems. *Journal of magnetic resonance* **2011**, *208* (2), 179–194.

(5) Kiryutin, A. S.; Sauer, G.; Yurkovskaya, A. V.; Limbach, H.-H.; Ivanov, K. L.; Buntkowsky, G. Parahydrogen allows ultrasensitive indirect NMR detection of catalytic hydrogen complexes. *The Journal of Physical Chemistry C* **2017**, *121* (18), 9879–9888.

(6) *Gaussian 16*; Gaussian, Inc.: Wallingford, CT, USA, 2016.

(7) Goodwin, D. L.; Kuprov, I. Auxiliary matrix formalism for interaction representation transformations, optimal control, and spin relaxation theories. *The Journal of chemical physics* **2015**, *143* (8).

(8) Redfield, A. G. On the theory of relaxation processes. *IBM Journal of Research and Development* **1957**, *1* (1), 19–31. Wangsness, R. K.; Bloch, F. The dynamical theory of nuclear induction. *Physical Review* **1953**, *89* (4), 728.

(9) Kuprov, I. Dissipative Spin Dynamics. In *Spin: From Basic Symmetries to Quantum Optimal Control*, Springer, 2023; pp 223–289.

(10) Natterer, J.; Greve, T.; Bargon, J. Orthodeuterium induced polarization. *Chemical physics letters* **1998**, *293* (5-6), 455–460.

(11) Kuprov, I. Incomplete Basis Sets. In *Spin: From Basic Symmetries to Quantum Optimal Control*, Springer, 2023; pp 291–312.

(12) Kuprov, I.; Hore, P. Chemically amplified 19F–1H nuclear Overhauser effects. *Journal of Magnetic Resonance* **2004**, *168* (1), 1–7.
